# Trending ticks: using Google Trends data to understand tickborne disease prevention

**DOI:** 10.3389/fpubh.2024.1410713

**Published:** 2024-06-13

**Authors:** Cheng-Xian Yang, Lauri M. Baker, Ashley McLeod-Morin

**Affiliations:** ^1^Department of Agricultural Education and Communication, University of Florida, Gainesville, FL, United States; ^2^UF/IFAS Center for Public Issues Education in Agriculture and Natural Resources, Gainesville, FL, United States

**Keywords:** Google Trends, health communication, online search, risk communication, ticks, tickborne disease, vector-borne diseases

## Abstract

**Introduction:**

Ticks and pathogens they carry seriously impact human and animal health, with some diseases like Lyme and Alpha-gal syndrome posing risks. Searching for health information online can change people’s health and preventive behaviors, allowing them to face the tick risks. This study aimed to predict the potential risks of tickborne diseases by examining individuals’ online search behavior.

**Methods:**

By scrutinizing the search trends across various geographical areas and timeframes within the United States, we determined outdoor activities associated with potential risks of tick-related diseases. Google Trends was used as the data collection and analysis tool due to its accessibility to big data on people’s online searching behaviors. We interact with vast amounts of population search data and provide inferences between population behavior and health-related phenomena. Data were collected in the United States from April 2022 to March 2023, with some terms about outdoor activities and tick risks.

**Results and Discussion:**

Results highlighted the public’s risk susceptibility and severity when participating in activities. Our results found that searches for terms related to tick risk were associated with the five-year average Lyme Disease incidence rates by state, reflecting the predictability of online health searching for tickborne disease risks. Geographically, the results revealed that the states with the highest relative search volumes for tick-related terms were predominantly located in the Eastern region. Periodically, terms can be found to have higher search records during summer. In addition, the results showed that terms related to outdoor activities, such as “corn maze,” “hunting,” “u-pick,” and “park,” have moderate associations with tick-related terms. This study provided recommendations for effective communication strategies to encourage the public’s adoption of health-promoting behaviors. Displaying warnings in the online search results of individuals who are at high risk for tick exposure or collaborating with outdoor activity locations to disseminate physical preventive messages may help mitigate the risks associated with tickborne diseases.

## 1 Introduction

Ticks carry and transmit infectious pathogens to humans and animals around the world, causing negative impacts on human health and the economy (1). The Centers for Disease Control and Prevention (2, 3) reported that approximately 500,000 people in the United States are diagnosed with tickborne diseases annually, propelling it to become an urgent public health issue. Ticks cause many infectious diseases and severe health problems (4). Species such as *Ixodes scapularis* (black-legged tick) and *Amblyomma americanum* (lone star tick) are distributed across various regions in the United States and can cause many infectious diseases and severe health problems (4). These species are known vectors of several tickborne diseases, including Lyme disease and Alpha-gal syndrome (5–7). Lyme disease leads to fever, headache, fatigue, and a rash. Untreated, it can affect joints, heart, and nerves. Alpha-gal syndrome, from lone star tick bites, causes allergic reactions to red meat, including hives, stomach pain, and anaphylaxis. Also, global changes, including climate patterns and intensified human activities, have increased tickborne diseases worldwide and in the United States, exacerbating their transmission and making them a growing public health concern (2, 8, 9). However, the seriousness of tickborne diseases is often underestimated. Regular forecasting and vigilant monitoring are required because early detection and treatment of the disease are important strategies in managing the public health threat posed by these diseases (10–12).

Additionally, the CDC (6) recommended that people who live in tick-prone areas and spend time outside pay more attention to taking preventive actions because they are more likely to be bitten by ticks and are at high risk for tickborne disease infections. People at high risk require focused attention to enhance communication efforts directed toward them. This targeted approach is designed to alleviate the burden of diseases and improve public health outcomes in tick-prone areas by implementing interventions that concentrate on modifying personal behaviors (2, 13).

The digital transformation in public health is revolutionizing intervention strategies, disease surveillance, and management through innovative technologies. Specific examples include wearable technology that enables real-time monitoring of health statistics, mobile applications that help manage chronic diseases, and artificial intelligence that improves the speed and accuracy of medical diagnosis (14, 15). In addition, with more and more people turning to the internet for health-related information, the landscape of health decision-making and behavior is rapidly evolving (16–18). More than 70 percent of people in the United States seek health information mainly through the internet, which is growing (19). New search tools and technologies in digital health are emerging to advance healthcare research (15, 20). Specifically, online channels can be one of the ways to access digital health information. Research aimed to learn how people talk about and perceive risks related to ticks and tickborne diseases on Twitter, which is now called X (21). The insights facilitate more effective communication strategies and content designs tailored for tick risk prevention, thereby contributing to improved public health outcomes. Recognizing the potential role of online communication in disseminating information about ticks and tickborne diseases, analyzing people’s online search behaviors regarding these risks can better promote the public adoption of health behaviors in the digital era.

The Health Belief Model (HBM), initially proposed by Becker in 1974 (22) and later refined by Janz and Becker in 1984, has been widely used to explain the health behavior change process. The HBM is built around six fundamental elements: the perceived severity and susceptibility associated with a health condition, the perceived benefits and barriers of adopting a recommended health behavior, cues to action (which serve as direct triggers for the behavior), and the confidence in one’s ability to adopt the behavior, known as self-efficacy (23, 24). According to the HBM, when individuals perceive both the severity of the potential consequences of vector-borne diseases and the susceptibility, and when they believe that the benefits of adopting preventive measures outweigh any perceived barriers, they are more likely to take proactive steps to protect themselves against vector-borne diseases (13, 25, 26). In addition, providing cues to action is important in motivating individuals to embrace healthier behaviors (27, 28). These cues can take the form of evidence or real-life experiences others share. Such prompts serve as catalysts, encouraging people to internalize the significance of prevention and reinforcing that these measures are effective and imperative for protecting their health.

Applying the HBM to interpret online search behaviors in public health revealed that awareness of disease severity and susceptibility enhances the likelihood of individuals taking preventive measures. For instance, analyzing the patterns of online searches related to HIV through big data can predict future infection risks, suggesting a correlation between search frequencies for HIV information and outbreak locations (29, 30). In addition, online search behaviors related to vector-borne diseases occurring during specific periods can serve as an effective tool for predicting, controlling, preventing, and reporting epidemic cases. For example, the incidence of mosquito-borne diseases such as dengue and yellow fever in tropical regions is closely correlated with the popularity of related search terms on online search engines (31, 32). Such as (1) symptoms associated with mosquito-borne diseases, (2) preventive measures, such as “mosquito repellent,” “mosquito net,” “vaccination,” (3) geographic locations or regions affected by mosquito-borne diseases, such as “tropical regions,” “subtropical climates,” (4) specific countries or cities where outbreaks occur, and (5) public health campaigns or initiatives related to mosquito-borne diseases, such as “mosquito control,” “vector control,” “public health interventions,” among others. Such digital footprints become powerful predictors of public health needs. Similarly, online searches about ticks and tickborne diseases may reflect public awareness and pinpoint potential outbreak areas and periods, effectively informing and guiding public health strategies. Research on online search results about tickborne diseases is still lacking, making a niche for our study to fill by exploring how digital behaviors correlate with public awareness and prevention efforts.

This study aimed to forecast potential tickborne disease risks through online search behavior. The CDC (2) emphasized the necessity and urgency of reducing tickborne disease risks, as nearly half a million people are diagnosed and treated for a tickborne disease each year. Taking measures to protect oneself and family members from tick bites is the best way to prevent tickborne diseases. These include using tick repellents, wearing long sleeves and pants, and performing regular tick checks after spending time outdoors, etc. (33). Also, through accurate symptom identification and timely intervention, early detection and treatment can significantly reduce the mortality rate associated with tickborne diseases (2, 34). By analyzing search trends in different regions and periods in the United States, this study identified those with potential tick-related risks and provided suggestions for communication strategies that promote people’s adoption of health behaviors to alleviate the dangers of tickborne diseases. Moreover, tick-related risks are most likely to occur in backyards, neighborhood green spaces, and public recreational lands (35). Recognizing that individuals engaging in outdoor activities face elevated tick-related risks (6), we utilized the insights from online search behaviors to analyze which outdoor activities are associated with higher levels of risk. Three research questions were proposed:

RQ1: Based on online search results, which regions (states) and months may have a higher risk of tickborne diseases?

RQ2: Based on online search results in different states, which outdoor activities are associated with both risks of tick bite and tickborne disease?

RQ3: Based on online search results at different times of the year, which outdoor activities are associated with both risks of tick bite and tickborne disease?

## 2 Methods

### 2.1 Research design

To address our research questions, we investigated individuals’ online search behaviors related to tick risks and juxtaposed these with real-world data and outdoor activities to discern patterns and associations. The rationale of our research design hinges on the idea that an increased volume of searches for specific terms within a particular region or time frame serves as an indicator that individuals in that context are encountering issues related to those terms (29, 30). Therefore, if a significant correlation exists between trends in tick-related terms searches and the incidence rates of tickborne diseases in corresponding locations and times, it indicates that people’s online search results effectively mirror real-world situations. This means a higher volume of online searches signifies greater exposure to tick risks. Such results can then be used to examine search trends for tick-related terms alongside specific outdoor activities. The approach allowed us to gage public awareness and perceived risks, contributing to a better understanding of how external factors and personal behaviors interplay in the context of tick exposure.

Google Trends was selected as a representative search engine to analyze people’s online search behavior because Google accounted for more than 90% of the search engine market share by all platforms, which is the most commonly used search engine (36). Google Trends is a free online search tool that allows users to see how frequently a search term or topic has been searched for on Google with a sample of Google web searches (37, 38). The tool allows users to compare the popularity of multiple search terms or topics and shows other topics people are also searching for when seeking a specific term or topic (38). Google Trends empowers individuals, businesses, and researchers to delve deeper into public interest on various topics, including products, events, and specific phenomena. This is achieved through data visualization in graphs and charts, allowing them to stay updated on evolving search behaviors and emerging trends in the digital landscape.

Google Trends provides a time series index that shows the frequency of search queries entered by users in a specific geographic location. The index is calculated based on query share, which is the total volume of searches for a specific term or topic divided by the total number of queries in that given geographic area during the examined time period (37). Google Trends employs a standardized scale to represent search query shares within a specified time frame. The highest share is assigned a value of 100, indicating the highest level of interest, while a value of 0 denotes search terms with low volume (37, 38). A low-volume term indicates that the term or topic has a minimal number of searches within the specified time period. This suggests that the term is not widely searched for or may not be of significant interest to the general population in the area during that time frame.

Google Trends data is predictable (39). This predictability is especially evident in specific terms or topics that exhibit regular fluctuations at certain periods throughout the year, with health-related subjects standing out for their high proportion of predictable inquiries. Therefore, with this publicly accessible tool, researchers can interact with the vast amounts of population search data and provide inferences between population behavior and health-related phenomena (18, 20). This capability offers a powerful lens through which to understand and address various health-related issues and trends within communities and populations.

### 2.2 Data collection and samples

This study collected the data through Google Trends in the United States from April 2022 to March 2023. This study can see trends over time by viewing a complete year of data. For the terms used for analysis, some associated with agritourism and outdoor activities (e.g., u-pick, hunting) were selected due to their common occurrence in grassy, brushy, or wooded areas, which can be habitats conducive to ticks (6, 40). Some terms about tick risks (e.g., Lyme disease, tick bite) from the CDC were also selected (5, 34). In addition, red meat allergy caused by lone star tick bites has recently attracted people’s concern (6, 21), so we added these terms. We tried to search with the term “Alpha-gal syndrome,” which refers to a severe allergic reaction that occurs after people eat red meat. The disease is associated with bites by the lone star tick. However, there is insufficient data for Google Trends to analyze due to too few searches for this word during the data collection period.

Google Trends accounts for variations in accents, spellings, and whether terms are in plural or singular forms, treating them as different (20), so a series of the same terms was used as the same topic. Regarding search input, users can search for multiple terms by combining them with “+” signs and can use quotation marks to indicate exact search phrases (41). Due to the search results of the tool being relative search volume, this research queries each word separately to know the search volume of each word in different regions and times to prevent the search volume of a single word from affecting other terms’ volume. Specifically, each word’s search volume is calculated independently so that each term can have a relatively high volume (100) in a location or period. All the topics and terms we used are in Table 1.

**Table 1:** Topics and terms for Google Trends searching.

Topic	Search term	Search method
Outdoor activity	U-pick	“you pick” + you-pick + “u pick” + u-pick + “you picks” + you-picks + “u picks” + u-picks
	Corn maze	“corn maze” + cornmaze + “corn mazes” + cornmazes
	Park	park + parks + “national park” + “national parks” + “state park” + “state parks”
	Hiking	hiking + “hiking trail”
	Hunting	hunting + “hunting lodge” + “hunting ranch” + “shooting preserve” + “game ranch” + “hunting lease”
Tick risks	Ticks	tick + ticks
	Tick bite	“tick bite” + tickbite + “tick attack”
	Tick repellent	“tick repellent” + “bug repellent” + “tick repel” + “bug spray”
	Lyme disease	“Lyme disease” + Lymedisease
	Meat allergy	“meat allergy”
	Lone star ticks	“lone star ticks” + “lone star tick”

### 2.3 Data analysis

After data collection, SPSS 29 was used for the descriptive analysis to see the trends of searching tick-related risks in different states and time periods. Bivariate Pearson Correlation analysis was used between the searching terms to determine the relationships between people’s online search about tick-related risks and real-world data and that with online search about outdoor activities. As of 2024, the available real-world data on tickborne diseases in the United States comes from the statistics on Lyme disease from the CDC (3). Therefore, we decided to average the existing data from the last 5 years (2018–2022), using the Lyme disease incidence rates by state and Lyme disease cases by week to smoothen short-term fluctuations and enhance reliability. We further analyzed the relationship between these figures and people’s online search behaviors.

### 2.4 Limitations

This study acknowledges limitations in its analytical tools. Firstly, despite a high internet penetration rate of 93% in the United States (42), it is crucial to consider digital inclusion issues. This includes the digital divide affecting marginalized and rural populations and overlooking those without internet access.

Besides, the data from Google Trends is a relative search volume, not an absolute value. This means that even though both terms have the highest search volume (100) in a specific region or time, there is still a difference in the absolute value of the two terms. To deal with this problem, researchers can put many words for analysis together in Google Trends, and it causes only one relatively highest search volume; that is, stronger comparability between different words can be seen. However, due to the difference in the absolute value of each word, for example, the number of searches for “ticks” is much higher than that of “lone star ticks,” if they are analyzed together, there may not be apparent differences in region and time for the latter because all relative volumes may be less than 1. Given this, this study decided to search and analyze the results of each word separately to avoid the mutual influence of the times between terms.

## 3 Results

### 3.1 Higher relative search volume about tick-related risks in different states and periods

This study first examined which states or time periods have more frequent searching behaviors about tick-related risks so as to infer potential risks in different dimensions. Results based on geographic location indicated that the first four terms about tick-related risks (ticks, tick bite, tick repellent, and Lyme disease) with higher relative search volume were mainly concentrated in the northeast United States. Specifically, states such as Maine, Vermont, New Hampshire, West Virginia, and Kentucky showed significant search interest, with relative search volumes consistently at or above 50. Maine and Vermont had all four terms with search volumes ranging from 78 to 100 and 89 to 100, respectively. New Hampshire and West Virginia also demonstrated high search volumes for all four terms, ranging from 65 to 78 and 53 to 82, respectively. While showing high search volumes for two of the four terms, Kentucky ranged from 56 to 59. Additionally, the terms “meat allergy” and “lone star ticks” with a higher search rate were also concentrated in states in the eastern United States. Missouri, Kentucky, Tennessee, Maryland, Virginia, and Oklahoma all had relative search volumes at or above 50. Missouri and Kentucky had search volumes ranging from 85 to 100 and 92 to 100, respectively. Tennessee had consistent search volumes of 82 for both terms. Maryland, Virginia, and Oklahoma also exhibited high search volumes, with values ranging from 75 to 80, 73 to 80, and 65 to 66, respectively. Based on the search results, people living in these areas are more likely to be exposed to ticks in their lives and thus have a higher probability of being exposed to tick-related risks. Table 2 shows the states with a higher relative search volume about tick risks.

**Table 2:** States in the United States with higher relative search volume about tick-related risk terms from Google Trends.

Ranking	Ticks	Tick bite	Tick repellent	Lyme disease	Meat allergy	Lone star ticks
1	ME (100)	VT (100)	VT (100)	ME (100)	MO (100)	KY (100)
2	VT (89)	ME (78)	ME (95)	VT (91)	KY (92)	DE (89)
3	NH (65)	NH (70)	NH (78)	WV (82)	TN (82)	MO (85)
4	WV (57)	WV (53)	KY (59)	NH (71)	MD (80)	AR (83)
5	KY (56)		WV (56)	RI (61)	VA (73)	TN (82)
6	AR (55)		AR (52)	CT (58)	OK (66)	VA (80)
7	MI (55)		IA (51)	PA (53)		MD (75)
8	MO (55)		PA (50)	MA (51)		KS (73)
9	KS (51)		NE (50)			WV (69)
10	PA (50)					OK (65)
11	CT (50)					ME (55)
12						NJ (51)
13						NE (51)

Google Trends standardizes the highest query share to 100, and states with relative search volume ≥ 50 have remained; AR = Arkansas; CT = Connecticut; DE = Delaware; IA = Iowa; KS = Kansas; KY = Kentucky; MA = Massachusetts; MD = Maryland; ME = Maine; MI = Michigan; MO = Missouri; NE = Nebraska; NH = New Hampshire; NJ = New Jersey; OK = Oklahoma; PA = Pennsylvania; RI = Rhode Island; TN = Tennessee; VA = Virginia; VT = Vermont; WV = West Virginia.

In our further analysis of the associations between the first four terms related to tick risks and the five-year average Lyme Disease incidence rates by state from CDC (3), we discovered a very strong and significant positive correlation between real-world data and the search terms volumes of “Lyme disease” (*r* = 0.90, *p* < 0.001), “tick bite” (*r* = 0.74, *p* < 0.001), and “ticks” (*r* = 0.71, *p* < 0.001). Additionally, a substantial relationship was also identified with “tick repellent” (*r* = 0.62, *p* < 0.001). The results suggested that online search behavior related to ticks and Lyme disease can serve as a valuable indicator of the real-world incidence of Lyme disease geographically. More specifically, according to real-world data provided by the CDC (3), the top five states with the highest five-year average Lyme disease incidence rates include Maine (ranked first in the United States with an incidence rate of 130.4), Vermont (ranked third with an incidence rate of 98.6), West Virginia (ranked fourth with an incidence rate of 77.0), and New Hampshire (ranked fifth with an incidence rate of 72.0). These findings corresponded with people’s online search trends in these States. Table 3 shows the bivariate correlations among online search terms and the data of Lyme Disease incidence rates by state.

**Table 3:** Bivariate correlation among online search terms from Google Trends and the data of five-year average Lyme Disease incidence rates by state in the United States.

	1.	2.	3.	4.	5.
	Ticks	Tick bite	Tick repellent	Lyme disease	Lyme Disease cases by states
1.	1				
2.	0.92**	1			
3.	0.77**	0.86**	1		
4.	0.86**	0.86**	0.72**	1	
5.	0.71**	0.74**	0.62**	0.90**	1

*p < 0.05, **p < 0.001; strength of relationship (43): 0.01–0.09 = Negligible, 0.10–0.29 = Low, 0.30–0.49 = Moderate, 0.50–0.69 = Substantial, > 0.70 = Very strong.

**Figure 1:**
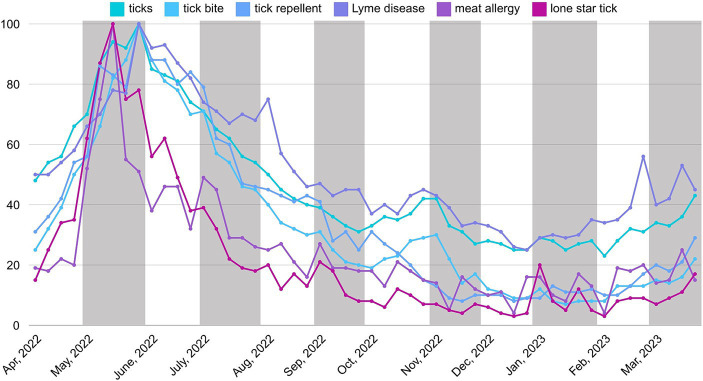
Relative search volume about tick-related risk terms in different time periods from April 2022 to March 2023.

On the other hand, when looking at different times within a year, terms about tick-related risks can be found to have higher search records during summer. The terms “ticks,” “tick bite,” “tick repellent,” and “Lyme disease” all experienced their peak search volumes in late May, specifically during the week of May 29. In contrast, the terms “meat allergy” and “lone star ticks” saw their highest levels of interest in mid-May, specifically during the week of May 15. The results showed that all terms have lower search volume in winter (Figure 1).

**Figure 2:**
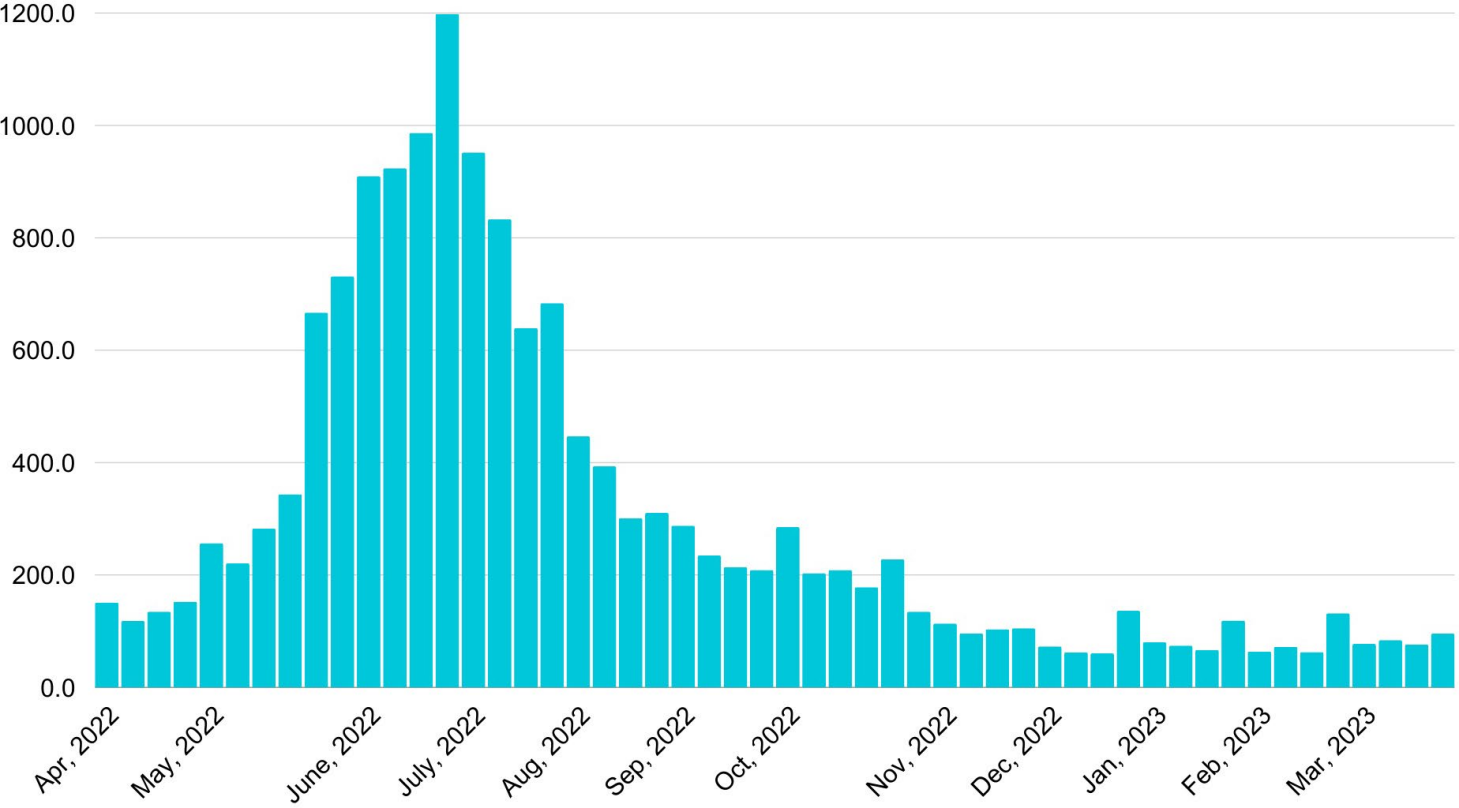
Five-year (2018–2022) average Lyme Disease cases by week in the United States [source: CDC (3)].

This study also analyzed the associations between the first four terms related to tick risks and the five-year average Lyme Disease cases by week from CDC (3). Search terms volumes of “ticks” (*r* = 0.68, *p* < 0.001), “tick bite” (*r* = 0.76, *p* < 0.001), “tick repellent” (*r* = 0.78, *p* < 0.001), and “Lyme disease” (*r* = 0.80, *p* < 0.001) show significant positive associations with the real-world data. These correlations provided a quantitative measure of the relationship between public search interest and the actual occurrence of Lyme disease, further validating the use of search data as a proxy for monitoring disease trends. More specifically, according to real-world data from the CDC (3), three of the top 5 weeks with the most Lyme disease cases occurred in June, and two occurred in July over a five-year average. Figure 2 shows the five-year average Lyme Disease cases by week in the United States. This showed that summer is the peak season for tick activity. The findings corresponded with people’s online search trends within a year period. Table 4 shows the bivariate correlations among online search terms and the data on Lyme Disease incidence rates by week.

**Table 4:** Bivariate correlation among online search terms from Google Trends and the data of five-year (2018–2022) average Lyme Disease cases by week in the United States.

	1.	2.	3.	4.	5.
	Ticks	Tick bite	Tick repellent	Lyme disease	Lyme Disease cases by week
1.	1				
2.	0.98**	1			
3.	0.96**	0.97**	1		
4.	0.93**	0.94**	0.94**	1	
5.	0.68**	0.76**	0.78**	0.80**	1

*p < 0.05, **p < 0.001; strength of relationship (43): 0.01–0.09 = Negligible, 0.10–0.29 = Low, 0.30–0.49 = Moderate, 0.50–0.69 = Substantial, > 0.70 = Very strong.

### 3.2 Correlations between search results about outdoor activities and tick-related risks in different states

For our RQ2, we expected to know the online search results about outdoor activities associated with tickborne disease risks in different geographic locations. The results among states indicated that search term volumes about “corn maze” are moderately related to “meat allergy” (*r* = 0.45, *p* < 0.001), and the “hunting” topic is moderately related to “ticks” (*r* = 0.34, *p* = 0.02). Regarding the search term volumes related to “hiking,” while it showed a negative correlation with “meat allergy” and “lone star ticks,” there existed a low positive correlation observed with “Lyme disease.” Such results reflected associations between specific search terms and highlighted the behavior patterns of individuals who engage in outdoor activities. In particular, the positive correlation between outdoor activity search terms and tick-related risk terms suggested that individuals who searched for outdoor activities were also inclined to look up terms related to tick-related risks.

**Figure 3:**
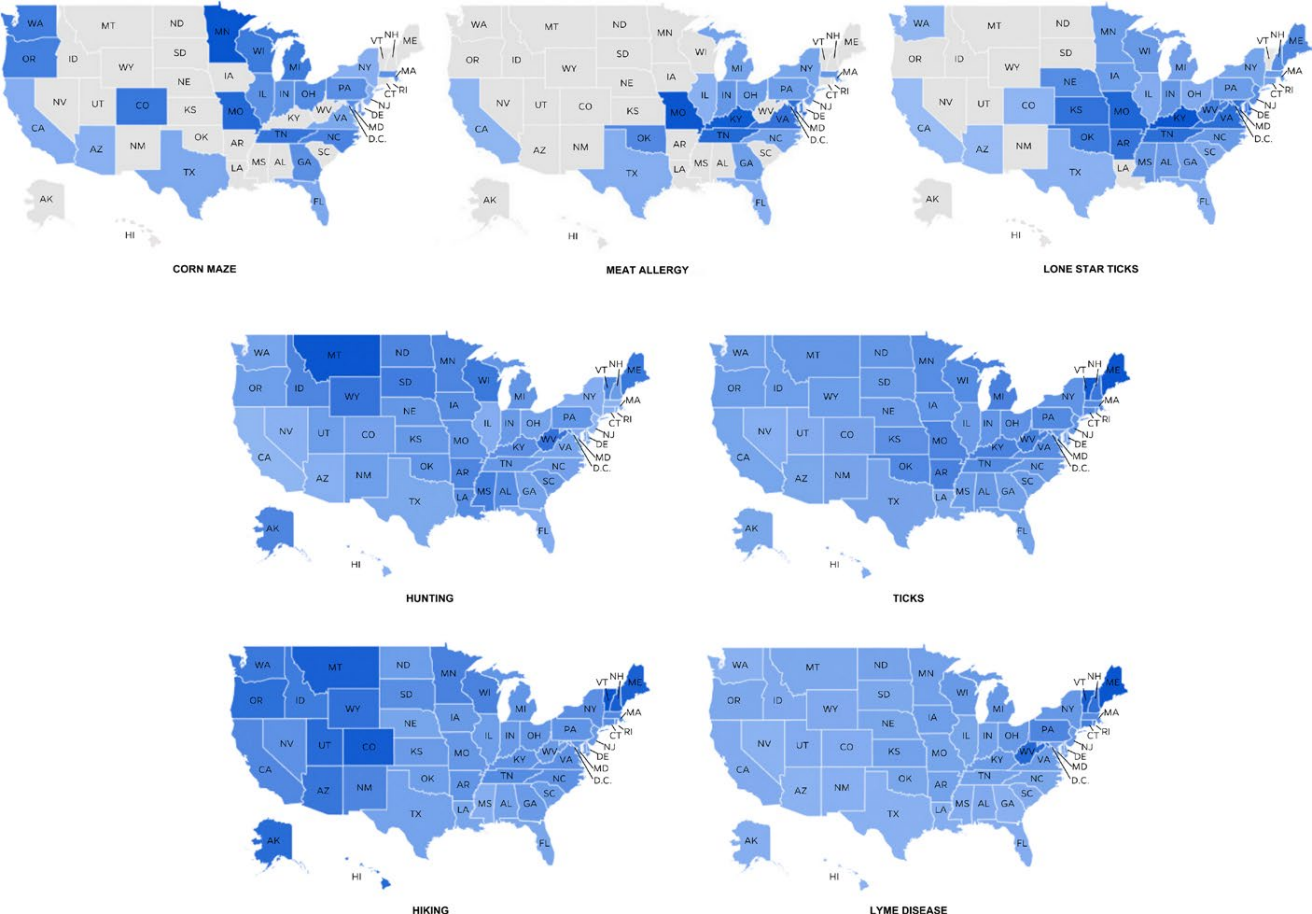
Data normalization of search terms between different states in the United States. The maps of the United States were moved from Google Trends with editing. AL = Alabama; AK = Alaska; AZ = Arizona; AR = Arkansas; CA = California; CO = Colorado; CT = Connecticut; D.C. = District of Columbia; DE = Delaware; FL = Florida; GA = Georgia; HI = Hawaii; ID = Idaho; IL = Illinois; IN = Indiana; IA = Iowa; KS = Kansas; KY = Kentucky; LA = Louisiana; ME = Maine; MD = Maryland; MA = Massachusetts; MI = Michigan; MN = Minnesota; MS = Mississippi; MO = Missouri; MT = Montana; NE = Nebraska; NV = Nevada; NH = New Hampshire; NJ = New Jersey; NM = New Mexico; NY = New York; NC = North Carolina; ND = North Dakota; OH = Ohio; OK = Oklahoma; OR = Oregon; PA = Pennsylvania; RI = Rhode Island; SC = South Carolina; SD = South Dakota; TN = Tennessee; TX = Texas; UT = Utah; VT = Vermont; VA = Virginia; WA = Washington; WV = West Virginia; WI = Wisconsin; WY = Wyoming.

Besides, of the many search terms for tick risks, only search term volumes about “lone star ticks” were related to “meat allergy,” which can better interpret our findings in our first results section. Table 5 shows the bivariate correlation results among searching terms between different areas. Through visual analysis, it can show data standardization of the terms in different regions more clearly (Figure 3).

**Table 5:** Bivariate correlation among online search terms between different states.

	1.	2.	3.	4.	5.	6.	7.	8.	9.	10.	11.
	U-pick	Corn maze	Park	Hunting	Hiking	Ticks	Tick bite	Tick repellent	Lyme disease	Meat allergy	Lone star ticks
1.	1										
2.	−0.04	1									
3.	−0.09	0.39**	1								
4.	0.29*	−0.14	−0.12	1							
5.	−0.01	−0.03	0.12	0.24	1						
6.	0.19	0.02	−0.19	0.34*	0.21	1					
7.	0.07	0.03	−0.22	0.16	0.17	0.92**	1				
8.	0.26	0.07	−0.21	0.03	0.11	0.77**	0.86**	1			
9.	0.05	−0.10	−0.20	0.22	0.28*	0.86**	0.86**	0.72**	1		
10.	−0.010	0.45**	−0.04	−0.21	−0.29*	0.18	0.20	0.14	−0.05	1	
11.	0.05	0.27	−0.03	−0.004	−0.38**	0.42**	0.43**	0.37**	0.17	0.64**	1

*p < 0.05, **p < 0.001; strength of relationship (43): 0.01–0.09 = Negligible, 0.10–0.29 = Low, 0.30–0.49 = Moderate, 0.50–0.69 = Substantial, > 0.70 = Very strong.

### 3.3 Correlations between search results about outdoor activities and tick-related risks in different time periods

Our last research question is to explore the online search results about outdoor activities associated with tickborne disease risks in different time periods. First, search term volumes of “u-pick,” “park,” and “hiking” have very strong associations with the frequency with which users searched for content related to the first four tick-related terms (*r* ≥ 0.71, *p* < 0.001) and substantial correlations (*r* ≥ 0.50, *p* < 0.001) with the terms “meat allergy” and “lone star ticks.” Across the United States, when people search for these outdoor activities, they are likely also searching for these pieces of risk information at the same time. We also found substantial negative correlations between search term volumes of “hunting” and all tick-related risk words. Table 6 shows the bivariate correlation results among searching terms between different time periods.

**Table 6:** Bivariate correlation among online search terms between different time periods.

	1.	2.	3.	4.	5.	6.	7.	8.	9.	10.	11.
	U-pick	Corn maze	Park	Hunting	Hiking	Ticks	Tick bite	Tick repellent	Lyme disease	Meat allergy	Lone star ticks
1.	1										
2.	0.001	1									
3.	0.85**	−0.25	1								
4.	−0.46**	0.47**	−0.71**	1							
5.	0.86**	−0.02	0.87**	−0.60**	1						
6.	0.71**	−0.22	0.77**	−0.59**	0.76**	1					
7.	0.77**	−0.17	0.78**	−0.52**	0.77**	0.98**	1				
8.	0.82**	−0.15	0.85**	−0.61**	0.84**	0.96**	0.97**	1			
9.	0.83**	−0.22	0.85**	−0.61**	0.81**	0.93**	0.94**	0.94**	1		
10.	0.54**	−0.16	0.68**	−0.53**	0.61**	0.86**	0.82**	0.83**	0.76**	1	
11.	0.50**	−0.23	0.63**	−0.57**	0.61**	0.93**	0.89**	0.88**	0.80**	0.93**	1

*p < 0.05, **p < 0.001; strength of relationship (43): 0.01–0.09 = Negligible, 0.10–0.29 = Low, 0.30–0.49 = Moderate, 0.50–0.69 = Substantial, > 0.70 = Very strong.

## 4 Discussion

### 4.1 Risks of tickborne diseases based on online search results

This study expected to predict Americans who are more likely to be exposed to high tick-related risks through people’s online search results and forecast potential relationships between outdoor activities and potential tick-related risks. Our first research question examined the regions and months that have a higher risk of tickborne diseases based on the online search results. First, based on the higher relative search volume results about tick-related risks in different states, several states in the northeastern United States (e.g., Maine, Vermont, New Hampshire, West Virginia, and Kentucky) have more search records, which may mean people living in these areas face more tick-related risks. Meanwhile, Maine (the five-year average Lyme disease incidence rate is 130.4), Vermont (98.6), West Virginia (77.0), and New Hampshire (72.0) have the highest five-year average Lyme disease incidence rates in the United States (3). Our analysis demonstrated a strong positive correlation between people’s online search results and regions experiencing outbreaks of tickborne diseases. Furthermore, while real-world data for Alpha-gal syndrome were unavailable for correlation analysis, our study still identified that the terms “meat allergy” and “lone star ticks” with a higher search rate had higher relative search volume in the eastern United States, which matches regions where lone star ticks live (2, 7, 11, 40). Therefore, higher search volume for specific terms coincides with areas where ticks and tick-borne diseases are endemic, further validating the use of search data to predict tick-related risks geographically.

In addition to geographic location, this study also looked at data from different time periods of the year. The results indicated the peak of all tick-related risk terms is from May to July, which overlaps with the time when ticks are more active in the United States. Ticks become more active in late spring to summer as temperatures rise and humidity levels increase (6, 33, 44, 45). This period also aligns with increased outdoor activities such as hiking and gardening, leading to higher exposure to ticks. Also, ticks are less active in winter due to the cold weather (45, 46); it can be found that the proportion of tick-related search volume is therefore reduced at this time. These environmental factors, combined with tick behavior, explained the spike in search interest from May to July and underscored the importance of public awareness during this critical period. Our correlation findings confirmed this observation, revealing substantial to very strong positive correlations between search terms and tickborne diseases.

Based on our results, we could infer that people’s online search behavior could reflect their exposure risks, both geographically and periodically (29, 30). This robust alignment between our findings and existing knowledge also underscored the consistency in the risk of tick bites. Ticks and the pathogens they carry can cause diseases that are a tangible part of people’s everyday lives, prompting them to seek information about associated risks actively. Individuals situated in high-risk environments or periods of vector-borne diseases are more inclined to conduct online searches to arm themselves with the necessary knowledge to mitigate these risks, resulting in an overall increase in search activity (31, 32). It reaffirmed that individuals face an increased risk in the northeastern regions (or eastern areas for lone star ticks), particularly during the summer months (3, 6, 7, 11, 40, 44). This correlation substantiates well-established patterns, indicating a higher incidence of tick-related risks in these contexts.

### 4.2 Outdoor activities and the risks of tick bite and tickborne diseases

Our second and third questions explored the correlations between search results about outdoor activities and tick-related risks in different states and time periods. The results can examine and infer possible associations between activity and tick risk. When a positive correlation exists between two terms, it may reflect that people are more likely to be exposed to tickborne diseases when they engage in that outdoor activity.

Based on the results of regional analysis, we inferred that people living in some states where corn mazes are prevalent in the eastern United States might be more concerned about suffering from Alpha-gal syndrome, which is caused by lone star ticks and causing meat allergies. When people hunt more in an area, they may be at higher risk of ticks; also, when people participate in outdoor activities related to hiking, they are more likely to be infected with Lyme disease from tick bites (35). Besides, Figure 2 provided a clearer illustration of the negative correlation between terms associated with “hiking” and those pertaining to “lone star ticks” and “meat allergy.” Apart from the Northeast region, states in the Midwest, such as Colorado, Montana, and Utah, exhibit a relatively high search volume for “hiking.” However, searches for terms like “lone star ticks” and “meat allergy” in these areas remain minimal. This observed pattern helps clarify the correlation.

On the other hand, looking at the results of time analysis within a year, we found that the terms “u-pick,” “park,” and “hiking” have strong associations with tick-related risks (*r* ≥ 0.50, *p* < 0.001), both Lyme diseases and Alpha-gal syndrome. This may imply that when people participate in these outdoor activities in the United States during the tick-active season, they may be more likely to be bitten by ticks (6, 33, 35). At the same time, they may also present higher tick-repellent needs. With these study results, people can further formulate and implement future risk communication strategies for specific online outdoor activity search behaviors to improve public health. Given that approximately 476,000 Americans are diagnosed and treated for Lyme disease each year (2) and that Alpha-gal syndrome has seen an increase in reported cases, especially in the southeastern and eastern United States (7), it highlighted the importance of public health communication strategies tailored to specific outdoor activity search behaviors. By understanding when and where people are more likely to engage in activities that expose them to ticks, public health officials can better target their messaging and interventions. This approach can help mitigate tickborne disease risks by promoting effective preventive measures such as using tick repellents, appropriate clothing, and timely checks for ticks after outdoor activities.

An interesting finding of this study is that when analyzing Americans’ search results periodically, “hunting” is negatively associated with all tick-related risk words. It is worth noting that white-tailed deer, a primary hunting target in North America, are most actively hunted from September to January (47). However, it is from October to November that the adult stage of the blacklegged tick, a key carrier of Lyme disease spirochetes, is most active (46). These ticks can predominantly infect white-tailed deer. Thus, we inferred that hunters may not be fully aware of this seasonal tick activity and may erroneously assume that ticks are primarily active during the summer months. This highlighted the importance of targeted risk communication efforts aimed at this specific group, ensuring they receive accurate information and take necessary precautions to protect themselves from tick risks.

### 4.3 The health belief model and practical application in public health

Research and educational strategies based on the HBM can improve the behavior and practices of the population in effectively implementing control measures to decrease the risks of vector-borne disease (13). The findings showed a higher perceived susceptibility and severity of risks in specific locations, times, and outdoor activities. We suggested that targeting people’s Google search results is a feasible and appropriate communication strategy to improve their health, which echoes previous suggestions on applying people’s search behaviors to design communication strategies and make information access easier and more efficient (17, 19). It can help health promoters predict when to provide more effective information to make people aware of risks and take preventive actions.

Google Trends results provide a preliminary situation about tick-related risks in the United States, and it can be used to discover potential risk information presentation to influence people’s behavior effectively. Designing communication contents as cues to actions will serve as triggers for people to take risk-prevention measures (27, 28). For example, through strategic partnerships with health organizations or search platforms, a targeted approach can be employed. When individuals residing in tick-prone regions conduct searches related to outdoor activities like “corn maze” or “hunting,” it is possible to do Google ads across the network for people who are using these terms as a prompt to action. This pop-up window could serve to alert them about the risks of tickborne diseases and provide education on preventive measures. Furthermore, this data suggests a potential collaboration with U-pick farms or corn mazes to disseminate prevention messaging on their platforms or at physical locations. This approach could be instrumental in mitigating risks associated with these activities.

In addition to improving people’s perceptions of risk, this can also lower the barriers to taking preventive behaviors. When people search for keywords such as “u-pick,” “park,” or “hiking,” the search results can remind them to wear trousers and stockings and prepare tick repellent in advance to improve people’s health outcomes through better online search results.

### 4.4 Conclusions and recommendations

To our knowledge, our study is the first to compare people’s online search behaviors with actual data to establish its potential in predicting tickborne diseases in the United States. Utilizing the HBM, we identified people’s susceptibility and severity regarding tick-borne diseases, allowing us to propose effective communication strategies to encourage the public to adopt health-promoting behaviors. Additionally, using big data and analytics, health promoters and organizations can tailor their communication and intervention efforts more accurately, making disease prevention efforts more effective overall.

This study is highly exploratory and practical. We discovered the predictive power of online search behavior for tickborne diseases, and it may be meaningful to infer the relationship of search behavior between outdoor activities and tick risks. However, future research is needed to further explore how good health promotion communication can be conducted with people at high risk of tick exposure to ensure they take risk-prevention behaviors. This study contributes to tick risk communication because we used a new analysis tool for online health search and provided some evidence for health promoters and organizations or search platforms to deliver health-related information to potential target audiences for risk communication.

The applications of digital tools in public health research, such as using Google Trends data in our study to forecast and analyze tickborne disease risks, provided valuable insight into how similar methodologies are employed in other disease contexts. For instance, researchers have utilized the analysis of online search behaviors to forecast new HIV diagnoses by examining the frequency of search terms associated with HIV (29, 30), as well as mosquito-borne diseases (31, 32), to anticipate future infection rates and identify outbreak locations. Our study demonstrated that search engine data can be a powerful predictor of public health trends and needs, which could be used for future studies with different disease management.

Our study highlighted the growing importance of digital tools in understanding and responding to public health challenges. By using real-time data and broad geographic information, public health professionals can better predict disease trends, tailor communication strategies, and implement more effective interventions tailored to the behaviors and needs of specific populations. This approach improves the immediacy and relevance of public health responses and facilitates a more proactive management of public health risks.

## Data availability statement

The original contributions presented in the study are included in the article/Supplementary material, further inquiries can be directed to the corresponding author.

## Author contributions

C-XY: Conceptualization, Data curation, Formal analysis, Methodology, Project administration, Visualization, Writing – original draft. LB: Conceptualization, Data curation, Funding acquisition, Resources, Supervision, Writing – review & editing. AM-M: Data curation, Visualization, Writing – review & editing.
